# Porcine Reproductive and Respiratory Syndrome Virus: Origin Hypothesis

**DOI:** 10.3201/eid0908.030232

**Published:** 2003-08

**Authors:** Peter G.W. Plagemann

**Affiliations:** * University of Minnesota, Minneapolis, Minnesota, USA

**Keywords:** porcine reproductive and respiratory syndrome virus, lactate dehydrogenase-elevating virus, virus evolution, arteriviruses

## Abstract

Porcine reproductive and respiratory syndrome is a serious swine disease that appeared suddenly in the midwestern United States and central Europe approximately 14 years ago; the disease has now spread worldwide. In North America and Europe, the syndrome is caused by two genotypes of porcine reproductive and respiratory syndrome virus (PRRSV), an arterivirus whose genomes diverge by approximately 40%. My hypothesis, which explains the origin and evolution of the two distinct PRRSV genotypes, is that a mutant of a closely related arterivirus of mice (lactate dehydrogenase-elevating virus) infected wild boars in central Europe. These wild boars functioned as intermediate hosts and spread the virus to North Carolina in imported, infected European wild boars in 1912; the virus then evolved independently on the two continents in the prevalent wild hog populations for approximately 70 years until independently entering the domestic pig population.

Several human and animal virus diseases, generally caused by RNA viruses, have emerged in the last 40 years (*1*,*2*). Some of these diseases are caused by preexisting viruses that have the capacity to infect alternate hosts under certain conditions (e.g., Ebola virus, hantavirus, and Nipah virus). Other diseases are caused by viruses that seem to have adapted to new hosts after accidental transspecies transmission. In addition to AIDS, which is caused by HIV, porcine reproductive and respiratory syndrome (PRRS) is a prime example of the latter class of diseases. PRRS, which affects domestic pigs, was first recognized approximately 14 years ago in North America (*3*) and in central Europe (*4*); this disease is now found worldwide and causes considerable economic losses in the swine industry (*5*). Initially, the disease was referred to as “mystery swine disease” until its cause was determined to be a positive-stranded RNA virus, designated porcine reproductive and respiratory syndrome virus (PRRSV), that together with murine lactate dehydrogenase-elevating virus (LDV), equine arteritis virus, and simian hemorrhagic fever virus, belongs to the family *Arteriviridae (**6*). However, the origin of PRRSV is still a mystery, especially since the European and North American PRRSV isolates cause similar clinical symptoms but represent two distinct viral genotypes whose genomes diverge by approximately 40% (*7*). The European and North American PRRSV prototypes are Lelystad virus (*4*) and VR-2332 (*3*), respectively. Retrospective serologic tests did not detect antibodies (Abs) to PRRSV in domestic pigs in Iowa and in Germany before the mid-1980s (*8*,*9*). The first seropositive pigs were discovered in herds in Iowa in 1985, Minnesota in 1986, and the former East Germany in 1988–1989.

Researchers have postulated that LDV and PRRSV, which are closely related, are derived from a common ancestor (*10*,*11*). I suggest that PRRSV is derived from LDV and that wild boars have functioned as intermediate hosts, based on the following observations.

The primary structural proteins of arteriviruses are the nucleocapsid (N) protein, the integral membrane/matrix (M) protein, and the primary envelope glycoprotein, GP5 (*10*–*12*). Both the M protein and GP5 of LDV and PRRSV seem to be triple membrane–spanning proteins whose short ectodomains of approximately 11 and 30 amino acids, respectively, are disulfide linked (*11*,*13*,*14*). The ectodomain heterodimer seems critical for the infection of macrophages, the primary host cell of all arteriviruses, perhaps playing a role in receptor interaction (*14*), but neither the GP5 ectodomain nor the M protein ectodomain appears to determine host cell tropism (*15*,*16*).

LDV was first isolated from tumor-bearing laboratory mice but later found to be endogenous in wild house mouse populations (*Mus musculus domesticu*s;*10**,**17*). LDV invariably causes a lifelong asymptomatic infection in mice that is recognized only by an elevation of plasma lactate dehydrogenase activity. The virus replicates cytocidally in a subpopulation of permissive tissue macrophages that clears excess lactate dehydrogenase from circulation. Persistent infection is maintained by replication in newly regenerated permissive macrophages and the escape from all host defenses. The single neutralization epitope located in the middle of GP5 ectodomain (Figure 1) is flanked in the common LDV isolates, represented by LDV-P (*10*), by two N-glycans that impair the immunogenicity of the epitope and render the viruses completely resistant to in vivo Ab neutralization (*18*,*19*). LDV, which is poorly transmitted between mice, is transmitted by biting and perhaps sexually but not via the respiratory route; oral transmission requires high amounts of virus (*10*,*20*). All LDVs isolated from tumor-bearing laboratory mice and wild house mice are genetically closely related to LDV-P. Nucleotide differences are largely found in the segments encoding the signal peptides of the glycoproteins or represent mostly translational silent substitutions (*21*,*22*), which indicates that LDV-P has attained close to evolutionary stasis (*23*).

**Figure 1:**
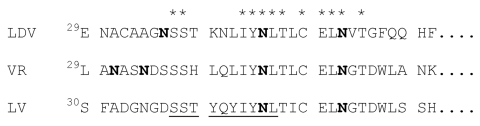
Amino acid comparison of the GP5 ectodomains of lactate dehydrogenase-elevating virus–P, porcine reproductive and respiratory syndrome virus VR-2332, and porcine respiratory and reproductive syndrome virus Lelystad virus (GenBank accession nos. U15146, U87392, and M96262, respectively). *Indicates amino identity. The neutralization epitope is underlined. N-glycosylation sites are in boldface letters.

In contrast to LDV, individual field isolates of both the European and North American PRRSVs exhibit great genome variability (e.g., phylogenetic analysis of open reading frame [ORF] 5, Figure 2). However, all PRSSV isolates are closely related to LDV, which is clearly indicated by amino acid comparisons of individual viral proteins. For example, the GP5 ectodomains of LDV, PRRSV VR-2332, and PRRSV Lelystad virus are collinear and contain a segment with approximately 70% amino acid identity (Figure 1). This segment contains the primary neutralization epitopes of LDV and of PRRSV (*24*,*25*), two highly conserved N-glycosylation sites and the Cys residue between them that is postulated to disulfide link the GP5 ectodomain to that of the M protein. The branching of the LDV sequence in the phylogenetic tree (Figure 2) in the line connecting the European and North American PRRSVs indicates that LDV is approximately equally related to both. This relationship is also indicated by the finding that some amino acids in all viral proteins are identical for LDV and VR-2332 but not Lelystad virus; vice versa, some amino acids are identical for LDV and Lelystad virus proteins only. For example, in the highly conserved segment of the GP5 ectodomain, two amino acids are identical in LDV and VR-2332 and one is identical in LDV and Lelystad virus (Figure 3; see also the discussion of the N-protein, and ORF1b protein).

**Figure 2:**
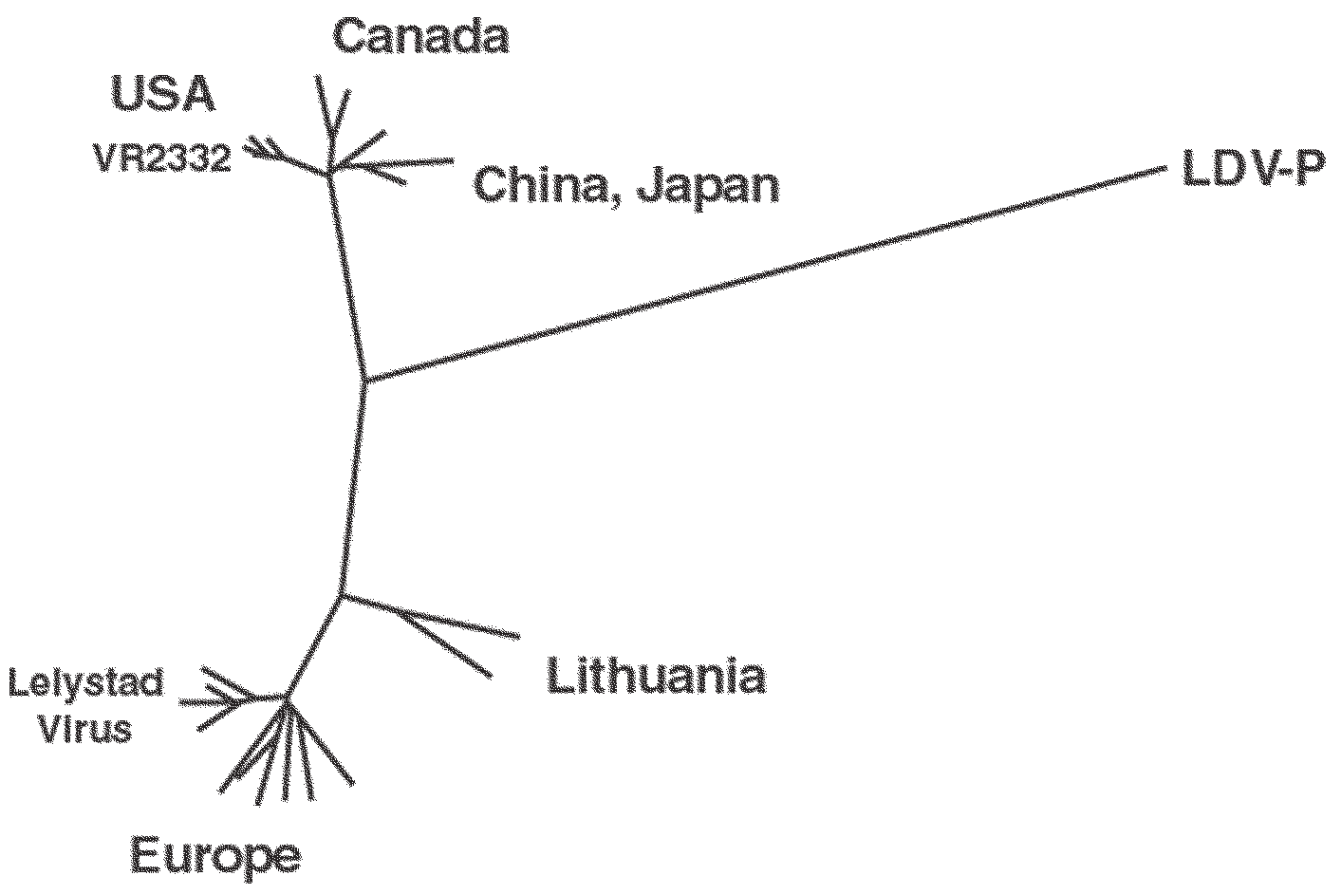
Phylogenetic tree of 432 nucleotide-long open reading frame (ORF) 5 segments of European and North American porcine reproductive and respiratory syndrome viruses and lactate dehydrogenase-elevating virus–P. The sequences correspond to nucleotide 97-526 of ORF5 of the European porcine reproductive and respiratory syndrome virus isolates (provided by T. Stadejek). LDV, lactate dehydrogenase-elevating virus.

**Figure 3:**
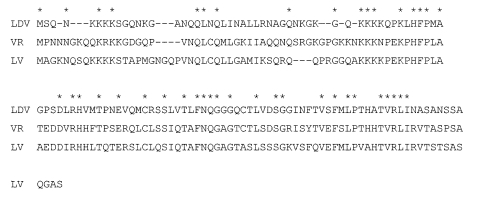
Amino acid alignment of the N-proteins of lactate dehydrogenase-elevating virus–P, porcine reproductive and respiratory syndrome virus VR2332, and porcine respiratory and reproductive syndrome virus Lelystad virus (115, 123, and 128 amino acids long, respectively). *Indicates identical amino acids

The genetic stability of LDV (which contrasts with the high variability of the PRRSV genomes typical for a new virus) and the relationship between the genome of LDV and those of the two PRRSV genotypes (Figure 2) both suggest that PRRSV has been derived from LDV. My hypothesis is that LDV was transmitted from an infected wild house mouse to a Eurasian wild boar (*Sus scrofa*) in central Europe sometime during the 19th century. At that time, wild boars were common throughout Europe, North Africa, and Asia (*26*). Such transmission would be rare because of species differences and the low transmissibility of LDV. The transmission may have occurred through oral means or wounds and involved a mutant form of LDV able to infect wild boars. The initial replication of this LDV mutant in and transmission between wild boars was likely slow until better host-adapted mutants were selected. The replication of the initially infecting virus might have been limited to tissue macrophages, as in mice. Where the initial virus transmission from wild house mice to wild boars occurred cannot be deduced from genome sequence comparisons. The ORF5 phylogenetic tree (Figure 2) suggests that the transmission could have occurred in Lithuania because the ORF5s of the Lithuanian isolates are more closely related to LDV than those of other European PRRSV isolates, but this relationship is not apparent in a phylogenetic analysis of ORF7 that encodes the N-protein. The initial infection of wild boars likely occurred in the eastern part of Germany (Sachsen-Anhalt), where the first PRRSV-seropositive pigs were discovered in Europe in 1988 and 1989 (*9*) and infected boars detected in this region in 1991 and 1992. From there the virus may have spread to the United States. Eurasian wild boars were introduced into the United States several times, but the primary introduction that became established occurred in 1912 when 14 wild boars (11 females and 3 boars) were released in a game preserve in the southwestern part of North Carolina in Hooper Bald (*26*). The origin of these wild boars is not entirely certain, but they probably came from the Harz Mountains in Sachsen-Anhalt. I postulate that one or more of these wild boars was infected with a PRRSV precursor virus and thus spread the virus to the United States. In 1912, feral pigs were widely prevalent throughout the United States (*26*). They were derived from domestic pigs that had escaped from farms, were intentionally released, or were free ranging. Soon after their importation, some wild boars escaped from the enclosed park; hybridization occurred between them and the feral pigs indigenous to the area (*26*). Most wild hogs now found in many U.S. states appear to be descendants of these hybrids. Wild boars from the North Carolina game farm were introduced into California in 1924, where they became well established. Wild boars were also released in Texas from 1930 to 1933 and became established there; these boars came from the San Antonio Zoo, but their original source is unknown (*26*).

After the introduction of PRRSV-infected wild boars in the United States in 1912, the virus would be expected to have evolved independently for 70 years in Europe and North America with the selection of mutants with better growth potential in the different wild hog populations on the two continents. Such independent evolution would explain the two distinct genotypes which, however, remain equally, but differently, related to LDV (Figure 2). Such selection of host environment–adapted mutants in this group of viruses is indicated by the rapid development of geographic clades of both the European and North American genotypes of PRRSV (*27*,*28*) (Figure 2). Such evolution must also have eventually involved the selection of mutants that efficiently replicate in alveolar macrophages, which is a property of PRRSV. This selection not only increased virulence of the viruses but also improved their replication potential in their hosts and transmission between the latter via the respiratory route.

Eventually, PRRSV variants infected pigs being raised domestically. Direct contact between wild hogs and domestic pigs in outdoor farms is not uncommon, and wild hogs have been intentionally introduced into existing swine herds. However, transmission could also have been mediated by products from hunted or slaughtered infected wild hogs. When and where this transmission might have occurred are unclear. Retrospective analyses found that approximately 1,400 serum samples collected in Iowa in 1980 lacked anti-PRRSV Abs. The first seropositive samples were detected in Iowa in 1985, and the earliest clinical symptoms were observed in herds in North Carolina, Iowa, and Minnesota (*29*). Thus, the first transmission from wild hogs to domestic pigs in the United States likely occurred in North Carolina, where wild hogs are numerous; the virus further evolved in the domestic pig population there and then spread to various midwestern states. By 1990, PRRSV had spread to 19 western, midwestern, and eastern states (*29*). In Europe, the first pigs with anti-PRRSV Abs were detected in East German herds in 1988 and 1989 (*9*) shortly before clinical symptoms of the disease were reported in herds in the central part of western Germany (*30*). A molecular clock of ORF3 suggests that the common ancestor of the European type of PRRSV infected domestic pigs around 1979 (*31*). This conclusion is not inconsistent with the relationship between the PRRSV strains in Europe and North America (Figure 2). The infection of the domestic pig populations on both continents may have occurred when the density of the wild hog and domestic swine populations increased considerably, which may have facilitated contact between them. Once virulent variants developed in the domestic pig populations, the spread of PRRSV has been extremely rapid on both continents as well as to other continents. Thus, pinpointing the location of the initial infection of domestic pigs is difficult.

Wild hogs with anti-PRRSV Abs have now been detected in both Europe and the United States. Two serum samples from 482 wild boars shot in 1991 and 1992 in Sachsen-Anhalt were found to have anti-PRRSV Abs by using the indirect immunoperoxidase monolayer assay. The PRRSV-positive boars were shot close to the former border to West Germany (*32*). Similarly, in northern France, 25 of 303 farmed wild boars tested in 1993 and 1994 and 8 of 603 shot in the same period were found to be seropositive by using the same serologic test or an indirect enzyme-linked immunosorbent assay (ELISA) (*33*), and 2 of 117 feral hogs tested in Oklahoma were seropositive in a commercial ELISA and a fluorescent Ab-staining test (*34*). However, no Abs were detected in 24 feral hogs killed in 1993–1994 in the Fort Riley Army Base in Kansas (*35*), in 44 wild boars shot in 1999 in Croatia (*36*), in 78 wild boars shot in 1999–2000 in Spain (*37*), or in >1,000 serum samples collected from wild boars in Eastern Europe (Stadejek, pers. comm.). The assumption is that seropositive wild hogs became infected by contact with infected domestic pigs (*33*), but no evidence exists to rule out that this infection may be an endogenous infection of wild boars that served as recent reservoir for the infection of domestic pigs. Regardless, the finding of PRRSV-seropositive wild hogs in both Europe and the United States indicates that wild hogs are susceptible to PRRSV infection. Wild hogs can also carry other viruses, such as those causing classical swine fever, Aujeszky’s disease, and pseudorabies; these viruses are considered an important potential source of infection of domestic pigs, especially in the case of classical swine fever.

In any case, the serologic tests available for the assay of anti-PRRSV Abs are likely inefficient or unsuitable for the detection of Abs to LDV-PRRSV intermediates that may be prevalent in wild hog populations. The tests are designed to detect anti-N-protein Abs, which are the primary Abs generated in PRRSV infected domestic pigs (*11*). Although the N-protein is a relatively conserved arterivirus protein, considerable amino acid differences exist between European and North American PRRSVs. For example, the N-proteins of Lelystad virus and VR-2332 exhibit only 60% amino acid identity (Figure 3). Several linear epitopes have been identified in the N-proteins of Lelystad virus and North American PRRSV, but they differ for the two PRRSVs and little serologic cross reaction occurs between them (*11*). Furthermore, these epitopes were identified by reaction with N-protein specific mouse monoclonal Abs (mAbs); only limited information is available on the immunogenicity of these epitopes in pigs. In addition, a common conformational epitope has been identified in the center of the N-protein (AA51-69), which exhibits 84% amino acid identity between Lelystad virus and VR-2332 (Figure 3). The corresponding segment of the LDV N-protein, however, exhibits much lower amino acid identity (42%) (Figure 3). Overall amino acid identity of the N-proteins of LDV, Lelystad virus, and VR-2332 is 36% (Figure 3); 15 additional amino acids are identical in LDV and VR-2332 and 10 amino acids in LDV and Lelystad virus, again indicating the close, but distinct, relationship of LDV with both PRRSV genotypes. The LDV/VR-2332 and LDV/Lelystad virus identical amino acids are primarily located in the C-terminal and N-terminal halves of the N-proteins, respectively. These amino acid differences between the LDV and PRRSV proteins may explain a lack of serologic cross-reaction between them; the same may be true for LDV-PRRSV intermediates. In addition, mice infected with LDV or immunized with inactivated virions do not generate Abs to the N-protein (*38*,*39*), apparently because the viral envelope or its proteins interfere with the immunogenicity of the N-protein since a mAb to the N-protein was generated by immunizing mice with isolated nucleocapsids (*40*). The primary Ab response of mice to LDV is to nonneutralization epitopes of GP5, which contrasts with the primary anti-N–protein Ab response of pigs or mice to PRRSV. These amino acid differences in Ab responses of pigs and mice may also apply to LDV-PRRSV intermediates and make it unlikely that their infection can be recognized by the available serologic tests for either LDV or PRRSV Abs. Furthermore, nothing is known about the prevalence of potential LDV-PRRSV intermediates in the wild hog population, the pathogenesis and course of infection of such viruses (or of PRRSV) in wild hogs, or the antiviral immune response of wild hogs.

An approach that is more suitable to detect LDV-PRRSV intermediates is reverse transcription–polymerase chain reaction (RT-PCR) with primers to highly conserved genomic sequences, for example, in a segment of the RNA polymerase domain upstream of the nidovirus characteristic SDD protein sequence (Figure 4). This nucleotide segment exhibits 53% identity between LDV, Lelystad virus, and VR-2332 (118/224 nucleotides; amino acid identity of the encoded protein segment is even higher, 75%). This segment contains short segments with complete nucleotide identity (Figure 4, stars); in addition, 25, 30, and 31 nucleotides are identical for LDV and VR-2332, LDV and Lelystad virus, and Lelystad virus and VR-2332, respectively, indicating again the close, but distinct, relationship between LDV and both Lelystad virus and VR-2332. My laboratory has previously used degenerate primer sets of this region designed on the bases of LDV, equine arteritis virus, and Lelystad virus sequence information (Figure 4) that detected not only the genomes of these three viruses but also those of VR-2332 and simian hemorrhagic fever virus, for which no sequence information was available at the time (*41*). The additional advantages of the RT-PCR approach are that this method can be readily applied to both serum and tissue samples and that the sequence of the PCR-amplified segment allows conclusions about the relatedness of the detected virus to existing viruses and the synthesis of specific gene probes for this virus. Also, degenerate primer sets can be designed to detect specific LDV-PRRSV intermediates. The same approach can be used to examine other species for the presence of arteriviruses.

**Figure 4:**
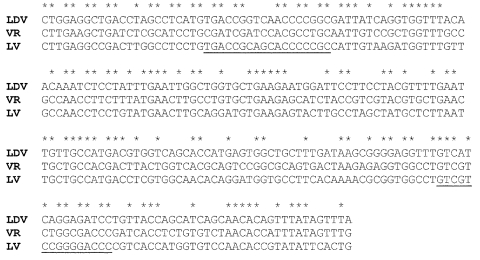
Nucleotide alignment of a segment of open reading frame (ORF) 1b of lactate dehydrogenase-elevating virus–P, porcine reproductive and respiratory syndrome virus VR-2332, and porcine reproductive and respiratory syndrome virus–Lelystad virus beginning at nucleotides 1169, 1165, and 1165, respectively. *Indicates identical nucleotides. Degenerate primer sets for polymerase chain reaction were previously made to the underlined segments (*41*).

My hypothesis on the origin of PRRSV encompasses all known facts but will be difficult, if not impossible, to prove, largely because of a lack of suitable materials for experimental investigation. My explanation of the theory serves to elicit interest in this subject and to encourage collaboration between investigators, especially in the search for stored or new materials that can be used to test the hypothesis.
